# Evaluation of a multiple ecological level child obesity prevention program: Switch^®^ what you Do, View, and Chew

**DOI:** 10.1186/1741-7015-7-49

**Published:** 2009-09-18

**Authors:** Douglas A Gentile, Greg Welk, Joey C Eisenmann, Rachel A Reimer, David A Walsh, Daniel W Russell, Randi Callahan, Monica Walsh, Sarah Strickland, Katie Fritz

**Affiliations:** 1 Department of Psychology, Iowa State University, Ames, IA, USA; 2 National Institute on Media and the Family, Minneapolis, MN, USA; 3 Department of Kinesiology, Iowa State University, Ames, IA, USA; 4 Department of Kinesiology, Michigan State University, East Lansing, MI, USA; 5 Des Moines University, Des Moines, IA, USA; 6 Institute for Social and Behavioral Research, Iowa State University, Ames, IA, USA

## Abstract

**Background:**

Schools are the most frequent target for intervention programs aimed at preventing child obesity; however, the overall effectiveness of these programs has been limited. It has therefore been recommended that interventions target multiple ecological levels (community, family, school and individual) to have greater success in changing risk behaviors for obesity. This study examined the immediate and short-term, sustained effects of the Switch program, which targeted three behaviors (decreasing children's screen time, increasing fruit and vegetable consumption, and increasing physical activity) at three ecological levels (the family, school, and community).

**Methods:**

Participants were 1,323 children and their parents from 10 schools in two states. Schools were matched and randomly assigned to treatment and control. Measures of the key behaviors and body mass index were collected at baseline, immediately post-intervention, and 6 months post-intervention.

**Results:**

The effect sizes of the differences between treatment and control groups ranged between small (Cohen's *d *= 0.15 for body mass index at 6 months post-intervention) to large (1.38; parent report of screen time at 6 months post-intervention), controlling for baseline levels. There was a significant difference in parent-reported screen time at post-intervention in the experimental group, and this effect was maintained at 6 months post-intervention (a difference of about 2 hours/week). The experimental group also showed a significant increase in parent-reported fruit and vegetable consumption while child-reported fruit and vegetable consumption was marginally significant. At the 6-month follow-up, parent-reported screen time was significantly lower, and parent and child-reported fruit and vegetable consumption was significantly increased. There were no significant effects on pedometer measures of physical activity or body mass index in the experimental group. The intervention effects were moderated by child sex (for fruit and vegetable consumption, physical activity, and weight status), family involvement (for fruit and vegetable consumption), and child body mass index (for screen time). The perception of change among the experimental group was generally positive with 23% to 62% indicating positive changes in behaviors.

**Conclusion:**

The results indicate that the Switch program yielded small-to-modest treatment effects for promoting children's fruit and vegetable consumption and minimizing screen time. The Switch program offers promise for use in youth obesity prevention.

## Background

Pediatric obesity continues to be a serious public health issue with trends indicating that the prevalence of childhood obesity will reach 24% in the USA by 2015 [1]. Reversing the pediatric obesity epidemic has been established as a critical priority [2]. Schools are the most frequent target for prevention and intervention programs because they reach the largest segments of the youth population and provide an available infrastructure and support [3-6]. Many school-based interventions aimed at modifying physical activity (PA) and diet have been conducted but the overall effectiveness of these programs has been limited [7,8]. One limitation is that many have not included families or communities.

Family components are critical for youth obesity prevention programs because parents directly and indirectly influence children's PA and nutrition behaviors at home and also dictate the physical and social environments that are available to their children [9]. Several studies have confirmed the importance of parenting behaviors and home environments in influencing children's nutrition and PA behaviors [10-12]. Despite the clear importance of families, family-based interventions (even those implemented through schools) have not been shown to be effective [13,14]. A possible reason for this is that these studies were conducted under isolated conditions and have not been well integrated into the schools and communities where families live, work and play. Several studies have demonstrated that home, school, and community environments interact over time to influence children's health and development [15,16], so it may prove more effective to integrate school and family interventions into broad community-based initiatives.

Social ecological models that target multiple levels of influence have been recommended to address the obesity epidemic [2,17]. Although a number of social ecological models exist to facilitate planning of multilevel interventions [18-21], there are few, if any, examples of studies that have implemented a multilevel intervention aimed at changing behavioral risk factors for obesity. This paper presents the main outcomes of Switch™, a family-, school-, and community-based intervention aimed at changing key behaviors (PA, television viewing/screen time, and nutrition) related to childhood obesity. Third through fifth graders were targeted to potentially avert or reduce the documented declines in fruit and vegetable consumption (FV) [22], PA [23], and increases in screen time (ST) [24,25] that occur during the late elementary and middle school years. Multiple behaviors were targeted in order to produce larger overall changes in energy balance. The American Dietetics Association (ADA) recently completed a comprehensive evidence analysis of behavioral factors that have been shown to be associated with childhood overweight [26]. Adequate evidence (Grade II) was found for five key behaviors (promoting PA, discouraging TV watching, decreasing dietary fat, decreasing sweetened beverages, and increasing FV). Consistent with these findings, the primary objectives of Switch were to: (1) increase the amount of PA; (2) reduce the amount of ST (television and video game time); and (3) increase FV consumption among children from the third grade to fifth grade.

## Methods

### Theoretical model

An established social ecological framework was used to guide the development of the Switch intervention [18,27]. Details of the application of the Switch model are described in detail in a separate publication [28]. The Switch program targets families as the primary leverage point. Parents influence eating behaviors by altering the types of food available in the home or restaurants, and the ways that food is prepared and consumed (for example, not in front of the TV) [9]. Parents also influence children's PA behavior (directly and indirectly) through efforts to encourage, facilitate, or promote activity and by preventing excessive amounts of inactivity [10-12]. Secondary leverage points included school-based reinforcement and incentives as well as community support. By providing integrated programming at three different levels (community, school, and family), it was theorized that this would have a greater impact than an intervention focused at only one level.

### Participants

All 10 elementary schools in Lakeville, MN and Cedar Rapids, IA, USA, participated in the study. These two school districts were approached due to the requirements of funding agencies. Schools were matched within district by enrollment and percent free/reduced-cost lunch and then randomly assigned to the experimental (three in Cedar Rapids and two in Lakeville) or control (three in Cedar Rapids and two in Lakeville) condition. Prior to participation, parents provided active written consent, and children provided assent.

**Figure 1:**
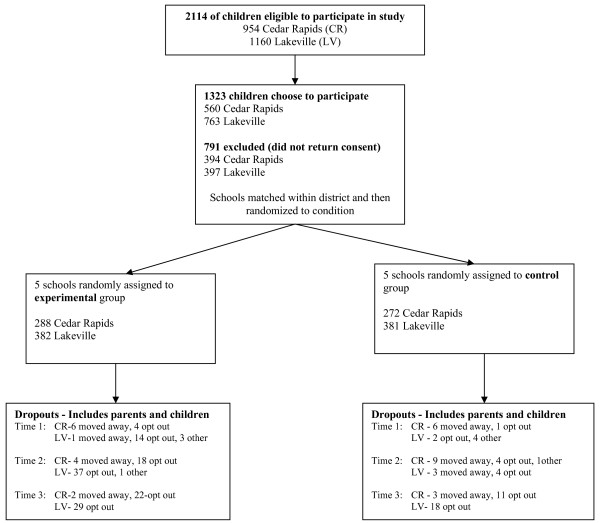
**Flow of participants through each stage of the program**.

A sample of 1,323 third (*N *= 430), fourth (*N *= 446), and fifth (*N *= 423) grade children returned consent forms (65% of total enrollment). Participation rates were similar between the experimental and control schools (673 out of 1019 children in experimental schools, 650 out of 1072 in control schools). Forty-seven percent were male (618 male, 704 female, 1 unknown) and most (90%) were White, which is representative of their communities. Figure 1 displays the flow of participants and recruitment details are described elsewhere [28].

Data were collected prior to implementation (baseline), immediately following the intervention (post-intervention), and 6 months post-intervention. Out of 1,323 consented families, 1,288 children (97%) provided data. Of those, 1,196 (93%) provided data at baseline, 1,156 (90%) at post-intervention, and 1,110 (86%) children at 6 months post-intervention. Data were provided by 1,076 children (84%) at both baseline and post-intervention, 1,029 (80%) at both baseline and 6-months post-intervention, and 992 (77%) at all three time points. A total of 1,150 (87%) parents provided data. Of those, 980 (74%) provided data at baseline, 916 (69%) at post-intervention, and 811 (61%) at 6 months post-intervention. Data were provided by 778 parents (59% of the total possible sample) at both baseline and post-intervention, 694 (52%) at baseline and 6 months post-intervention, and 631 (48%) at all three time points. Although the percentages varied between waves of data collection, over 80% of parent surveys were completed by mothers, between 7% and 11% were completed by fathers, and the rest were completed by both parents together.

The study was approved by the University of Minnesota Institutional Review Board in accordance with the Declaration of Helsinki and the 'Ethical Principles of Psychologists and Code of Conduct' [29]. It is registered as a clinical trial with ClinicalTrials.gov (NCT00685555).

### *Overview of the Switch Intervention*

The Switch program promoted healthy active lifestyles by encouraging students to 'Switch what you Do, Chew, and View'. The specific DO, VIEW, and CHEW goals were to be active for 60 minutes or more per day, to limit total ST to 2 hours or fewer per day, and to eat five fruits/vegetables or more per day. The intervention utilized overlapping behavioral and environmental strategies employed at multiple ecological levels, and is described in detail elsewhere [28].

The community component was designed to promote awareness of the importance of healthy lifestyles and the prevention of childhood obesity in the targeted communities, and included paid advertising (for example, billboards) and unpaid media emphasizing the key messages. Note that both experimental and control participants may have been exposed to the community component. The school component was designed to reinforce the Switch messages and facilitate the family component of the intervention. Teachers were provided with materials and ways to integrate key concepts into their existing curricula. Teachers were not required to participate, since the study was not designed as a school-based (curricular) intervention. Control schools did not receive any school materials. The family component was designed to provide parents (and children) with materials and resources to facilitate the adoption of the healthy target behaviors. Monthly packets containing behavioral tools were provided to assist parents and children in modifying their behaviors. Control families were recruited similarly to experimental families, but received no materials other than the surveys (families were not told if they were in the experimental or control conditions).

Data were collected from multiple informants. Baseline assessments (October 2005) included children wearing pedometers for 1 week, completing surveys in classrooms, and having anthropometric measurements taken by school nurses. Parents and teachers also completed surveys. The same measures were conducted immediately post-intervention (May 2006). To measure the maintenance of effects, data were collected approximately 6 months after the end of the intervention (November 2006).

### Measures

#### Physical activity

Habitual PA was assessed by a pedometer (Digiwalker 200-SW). Accuracy was assessed by having participants walk 10 steps and pedometers that were off by more than 1 step were replaced. Children were instructed to wear the pedometer for 7 consecutive days and to record the time they put the monitor on and off as well as the number of steps accumulated each day. At least 4 days' of data (3 weekdays and 1 day at the weekend) with at least 10 hours/day were required to be considered compliant (63% of children were compliant at baseline, with compliance dropping to 38% at later measurements). The classification of meeting physical activity goals was determined using two published recommendations. The Vincent and Pangrazi [30] recommendation is a norm-referenced criterion (recommending a minimum of 13,000 and 11,000 steps/day for boys and girls, respectively), and the Tudor-Locke is a criterion-referenced cut-point [31] (15,000 and 12,000 steps/day for boys and girls, respectively).

#### Anthropometry

Standing height and weight were measured by trained school nurses using standardized procedures [32]. Body mass index (BMI) was calculated as mass, kg/height, m2. Age- and sex-specific reference values were used to categorize weight status (normal weight, 5th to 84th percentile; overweight, 85th to 94th percentile; obese, 95th percentile or higher) of the participants [33].

#### Screen time

Time spent viewing TV and playing video games was assessed (independently) by both parents and children, using methods that have been used reliably with parents [34] and children [35,36]. Time spent on TV and video games was summed to create weekly ST (hours/week).

#### Fruit and vegetable consumption

Parents and children reported on children's FV consumption with items adapted from the National Youth Risk Behavior Survey [37]. The items evaluated the child's frequency of drinking 100% juice, sugared drinks, eating fruit, green salad, carrots, and other vegetables. Parents reported consumption over the previous 7 days and children reported for the previous day.

#### Perceptions of change at post-intervention

At the conclusion of the program, children and parents in the experimental group were surveyed about noticeable effects of the program on ST, FV, and PA. For each behavior measured, participants reported on a five-point verbally anchored scale the degree to which they perceived their family changed behaviors (ranging from 'a lot more' to 'a lot less').

Parents and children also reported how much they had participated in the various aspects of the program at post-intervention (11 items for parents, 9 items for children, for example, 'How much did your family use the Switch activity jar?'). Reports of parents and children were found to be significantly correlated (*r *= 0.47); we therefore standardized scores on each measure and a family involvement score was calculated by averaging parent and child ratings. This composite measure of family involvement allowed for testing whether involvement modified the effect of the intervention, as it could be hypothesized that the effect of the program would be greatest for families who were most involved. High and low involvement groups were created by a median split.

### Data analyses

Descriptive statistics were calculated for anthropometric, ST, FV, and PA variables at baseline grouped by experimental and control school and sex. There were no group differences on the primary outcome measures at baseline except for child reporting of FV, with children in the experimental group reporting higher FV (*P *= 0.04; see Table 1). These findings confirm that the matching and random assignment of schools to the treatment and control conditions were generally successful in equating the two groups.

**Table 1:** Descriptive characteristics of the sample on the outcome measures at baseline.

		**Total sample**	**Experimental schools**			**Control schools**		
		
**Variable**	***N*** **Total = 1,323^a^**		**Total** ***N *= 685**	**Males** ***N *= 301**	**Females** ***N *= 383**	**Total** ***N *= 674**	**Males** ***N *= 337**	**Females** ***N *= 336**
Age (years)	1,283	9.6 (0.9)	9.6 (0.9)	9.7 (0.9)	9.6 (0.9)	9.6 (0.9)	9.6 (0.9)	9.6 (0.9)
% Female	1,323	53.0%	56.0%	---	---	49.6%	---	---

**Anthropometric data**								

Height (cm)	1,286	138.2 (7.9)	138.5 (7.9)	138.8 (7.7)	138.2 (8.1)	138.0 (7.9)	138.6 (7.7)	137.4 (8.0)
Weight (kg)	1,281	35.6 (9.3)	35.6 (9.2)	35.4 (8.8)	35.8 (9.5)	35.6 (9.5)	36.2 (9.3)	34.9 (9.6)
Body mass index (kg/m^2^)	1,279	18.4 (3.4)	18.4(3.3)	18.2 (3.2)	18.5 (3.4)	18.5 (3.5)	18.6 (3.4)	18.3 (3.6)
% Overweight	1,279	19.1	18.8	21.5	16.1	19.5	16.4	21.9
% Obese	1,279	8.0	7.7	7.4	8.1	8.4	6.6	9.7

**Screen time **(hours/week)								

Child report	1,161	29.6 (23.5)	28.6 (22.5)	34.0 (24.6)	24.0 (19.4)	30.6 (24.4)	36.1 (27.4)	25.1 (19.5)
Parent report	980	22.1 (12.8)	20.7 (12.7)	22.0 (12.5)	19.7 (12.8)	23.3 (12.8)	26.1 (14.1)	20.6 (10.9)

**Fruit and vegetable consumption**								

Child report (servings/day)	1,101	4.5 (3.1)	4.9 (3.2)	4.5 (3.1)	5.3 (3.3)	4.1 (2.9)	4.1 (3.2)	4.0 (2.5)
Parent report (servings/week)	961	24.1 (13.5)	25.4 (14.1)	24.9 (14.0)	25.7 (14.2)	23.0 (12.8)	22.8 (12.9)	23.2 (12.7)

**Physical activity**								

Pedometer (steps/day)	826	11,665 (3,096)	11,735 (3,197)	13,058 (3,555)	10,861 (2,544)	11,594 (2,993)	12,412 (3,210)	10,832 (2,557)

^a^Sample sizes vary due to missing data.

The primary tests examining differences between baseline and post-intervention and baseline and 6 months post-intervention were conducted using hierarchical multilevel regression analyses to take into account the nested nature of the data (families are nested within schools). An analysis of variance was conducted to test for differences between the schools on the outcome measures at baseline. School had a significant effect for all six outcome variables, explaining from 1% to 11% of the variance in each. Because schools explained a significant amount of variance hierarchical analyses were required and, therefore, subsequent analyses of differences between the treatment and control groups employ variance due to school as the error term in the analyses. This adjustment reduces the degrees of freedom (df) from over 1,300 to 9, but is necessary due to the variability by school and the fact that participants were randomized at the school level. Baseline values were used as covariates to control for any differences between participants on these variables prior to the intervention. This also controls for the observed difference between groups for the child reporting of FV. Further analyses were conducted to provide a more comprehensive evaluation of the intervention. One set of analyses examined whether or not the child's sex moderated the effectiveness of the intervention. These analyses examined whether the intervention had a greater impact for boys or girls. Another set of analyses examined whether family involvement in the programming moderated treatment effects. These analyses specifically examined whether effects were stronger for participants that were highly involved in the program. A final set of analyses tested for moderation of treatment effects by weight status. These analyses specifically examined whether the effects were stronger for overweight or obese youth compared with normal weight youth. As we had directional hypotheses, all reported tests are one-tailed. Effect sizes (ES) were also calculated using Cohen's *d *as a measure of effect size that reflects the magnitude of the difference between groups in standard deviation (SD) units. Cohen's *d *is computed by subtracting the average score for the control group (M_C_) from the average score for the treatment group (M_T_), and then dividing the difference by the SD on the outcome measure for the sample. We used the standard criteria for ES of small (*d *= 0.20), medium (*d *= 0.50), and large effects (*d *= 0.80) [38]. We employed these criteria along with the results of the statistical tests in evaluating the impact of the Switch program. Data analysis was conducted using SAS and SPSS.

## Results

Characteristics of the sample at baseline are shown in Table 1. The average BMI was 18.4 kg/m^2 ^(SD = 3.4, range 12.4 to 35.4 kg/m^2^). Overall, 19% of children were classified as overweight and 8% were obese. There were significant differences between boys and girls on child-reported and parent-reported weekly ST (*t *(1,144) = 6.35, *P *< 0.001, and *t *(976) = 5.69, *P *< 0.001, respectively). This was due primarily to video game play. By child report, boys and girls were not significantly different on the number of fruits and/or vegetables they had eaten the previous day. Based on national guidelines (five servings of fruits and vegetables per day) approximately 44% of the children met the recommended guidelines. By parent report, girls ate almost two more servings of fruits and vegetables per week compared with boys (*M *= 21.7 and 23.4 for boys and girls, respectively; *t *(957) = 2.22, *P *< 0.05). Only 17% of children met the recommended guidelines of at least five servings a day. As expected, boys tended to have greater step counts than girls (*t *(823) = 9.01, *P *< 0.001). Depending on the recommendation used, between 28% and 43% of children achieved the recommended level of PA. Boys and girls were not significantly different on meeting the Vincent and Pangrazi recommendation (41% and 45%, respectively), but were significantly different on meeting the stricter Tudor-Locke recommendation (23% and 31%, respectively; χ^2 ^= 7.7, df = 1, *P *< 0.01).

### Treatment effects on key variables

Tables 2 and 3 display immediate post-intervention and 6-months post-intervention effects, respectively. Analyses compare experimental and control schools, controlling for baseline scores on each outcome measure. The effect sizes ranged from small (Cohen's *d *= 0.15 for BMI at 6 months post-intervention) to large (1.38; parent report of ST 6 months post-intervention). There was a significant difference in parent-reported ST at post-intervention, and this effect was maintained at 6 months post-intervention (a difference of about 2 hours/week). Child-reported ST was also lower in treatment groups at 6 months post-intervention, but the effect was not significant (although it did achieve a moderate effect size). There were significant increases in parent-reported FV consumption at post-intervention and 6 months post-intervention. The increase for child-reported consumption was marginally significant at post-intervention and was significant at 6 months post-intervention. PA, as measured by pedometer, at post-intervention and 6 months post-intervention was not statistically significant, although the students in the treatment schools accumulated an average of about 350 more steps per day (Cohen's *d *= 1.83, large effect). Despite a lack of statistical significance, these differences are in the expected direction. At both post-intervention and 6 months post-intervention, the mean BMI values were not significantly different between the treatment and control groups.

**Table 2:** Differences between experimental and control schools at immediate post-intervention

**Variable**	**Experimental schools** **(mean standard error)**	**Control schools** **(mean standard error)**	***t *(8)**	**Cohen's *d***
**Screen time**				

Child report (hours/week)	32.5 (0.6)	31.2 (1.0)	.26	.69

Parent report (hours/week)	22.8 (0.7)	24.6 (0.3)	-2.15^a^	1.26

**Fruit and vegetable consumption**				

Child report (servings/day)	4.4 (0.2)	4.2 (0.1)	1.78^b^	.52

Parent report (servings/week)	24.9 (0.7)	22.6 (0.4)	2.69^a^	1.36

**Physical activity**				

Pedometer (steps/day)	12,250 (260)	11,870 (232)	.91	1.83

**Body mass index**				

Body mass index (kg/m^2^)	19.0 (0.02)	19.0 (0.03)	.05	.38

Analyses compare experimental and control school with hierarchical multilevel regressions, controlling for baseline levels.

^a^Differences between experimental and control schools significant at *P *< 0.05, one-tailed; ^b^differences marginally significant at *P *< 0.06, one-tailed.

**Table 3:** Differences between experimental and control schools at 6-months post-intervention

**Variable**	**Experimental schools** **(mean standard error)**	**Control schools** **(mean standard error)**	***t *(8)**	**Cohen's *d***
**Screen time**				

Child report (hours/week)	27.8 (0.8)	29.1 (0.9)	-1.33	.67

Parent report (hours/week)	23.7 (0.5)	25.7 (0.5)	-2.06^a^	1.38

**Fruit and vegetable consumption**				

Child report (servings/day)	4.1 (0.2)	4.0 (0.1)	2.32^a^	.26

Parent report (servings/week)	22.5 (0.7)	21.3 (0.3)	1.93^a^	1.01

**Physical activity**				

Pedometer (steps/day)	11,442 (425)	11,231 (321)	.60	.26

**Body mass index**				

Body mass index (kg/m^2^)	19.4 (0.1)	19.5 (0.1)	-1.13	.15

Analyses compare experimental and control schools with hierarchical multilevel regressions, controlling for baseline levels.

^a^Differences between experimental and control schools significant at *P *< 0.05.

### Moderating variables

#### Sex

We examined whether the treatment effects were modified by child sex. These analyses examined the effect of treatment program, sex, and the program by sex interaction on the outcome measures, controlling for baseline values. Although boys had higher total ST than girls (due to more video game play), the interactions between sex and treatment were non-significant. In contrast, the sex by treatment interaction on child-reported FV was significant at post-intervention (*t *(961) = 2.84, *P *< 0.01) and 6 months post-intervention (*t *(907) = 1.99, *P *< 0.05). At both times, the interaction was due to girls in the treatment group reporting greater FV consumption (post-intervention *M *= 5.0, 6 months post-intervention *M *= 4.5) than girls in the control group (post-intervention *M *= 4.2, 6 months post-intervention *M *= 3.6). For PA, boys had higher step counts than girls at both post-intervention and 6 months post-intervention, but the interaction with treatment was significant at post-intervention, *t *(717) = 2.48, *P *< 0.05, with the program having a significant effect on girls (treatment *M *= 12,105 steps/week, control *M *= 11,180). For BMI, there was a significant sex by treatment interaction at 6 months post-intervention, *t *(1083) = 2.19, *P *< 0.05, with boys in the treatment group having a lower BMI (*M *= 19.1 kg/m^2^) than boys in the control program (*M *= 19.4 kg/m^2^).

#### Level of family involvement

We examined whether the treatment effects were modified by level of involvement, hypothesizing that the effects of the Switch program would be largest for families who were most highly involved. Significant interactions were found with child-reported FV consumption at post-intervention (*t *(460) = 4.61, *P *< 0.001) and at 6 months post-intervention (*t *(423) = 2.37, *P *< 0.05), and for parent-reported FV at post-intervention (*t *(340) = 3.05, *P *< 0.01), controlling for baseline values. Greater consumption of FV was reported by children who were highly involved (defined by median split) in the treatment program (post-intervention *M *= 5.3, 6 months post-intervention *M *= 4.7) than for children who were less involved in the treatment program (post-intervention *M *= 4.1, 6 months post-intervention *M *= 4.1).

#### Weight status

We examined whether the treatment effects were modified by child weight, hypothesizing that children who were classified as obese might benefit more from the program. Obese children reported higher ST at post-intervention (*M *= 35.5, controlling for baseline ST) than overweight (*M *= 30.9) or normal weight (*M *= 29.6) participants, *t *(1005) = 2.54, *P *< 0.05. This relation was moderated by a statistically significant interaction, *t *(1003) = -2.47, *P *< 0.05, with obese children showing the largest difference in ST between the treatment (*M *= 30.2) and control (*M *= 40.8) groups. Thus, the positive effect on child-reported ST was greatest for obese children.

### Treatment effects - participant perceptions

Table 4 documents perceived changes due to participation in the Switch program. One-third of children reported that they were watching less TV and/or videos and 23% felt that they were spending less time playing video games since starting the Switch program. Parents' perceptions were more positive with 44% reporting that their children watched less TV and/or videos and 31% reporting that their children played fewer video games. Almost half of children and parents reported that children ate more fruits (49% and 44%, respectively) and vegetables (39% for both children and parents) since beginning the program. About two-thirds (62%) of children and 34% of parents reported that children were more active since beginning the program.

**Table 4:** Perceptions of changes since participating in Switch (experimental participants only)

**Since starting the Switch program**	**A lot more**	**A little more**	**About the same**	**A little less**	**A lot less**
**Screen time**					

Do you watch more or fewer TV/Videos?	7%	8%	51%	34%	0%

Do you play more or fewer video games?	9%	13%	55%	23%	0%

Does your child watch more or fewer TV/Videos?	0%	1%	55%	40%	4%

Does your child play more or fewer video games?	0%	2%	67%	23%	8%

**Fruit and vegetable consumption**					

Do you eat more or fewer fruits?	22%	27%	47%	4%	0%

Do you eat more or fewer vegetables?	16%	23%	56%	5%	0%

Does your child eat more or fewer fruits?	5%	39%	56%	0%	0%

Does your child eat more or fewer vegetables?	3%	35%	61%	1%	0%

**Physical activity**					

Are you more or less active?	29%	33%	35%	3%	0%

Is your child more or less active?	4%	30%	66%	0%	0%

## Discussion and Conclusion

The goal of the present research was to determine whether a multilevel intervention conducted in two states could modify key behaviors related to overweight or obesity. The novel aspect of the study was the community-, school-, and family-based approach to modify nutritional and activity behaviors. In general, baseline data indicated that these children were similar to national averages for this age group. The intervention yielded encouraging results despite the relatively short-term length of the intervention, the relatively small number of schools, and the naturalistic design. The experimental group showed significant differences from the control group on both ST and FV consumption at both the immediate post-intervention assessment and at the 6-month follow-up assessment, controlling for baseline values. The results for selected variables were moderated by child sex, family involvement, and child BMI. The intervention was designed to influence three behaviors that together can have an impact on BMI over the long term and was not expected to have a short-term direct effect on BMI. It is likely that longer-term studies would be needed to document changes in BMI resulting from changes in nutritional and activity behaviors.

The effect sizes reported here range from small to large and are similar in magnitude to other published intervention studies [39-41]. Although modest, these results are not trivial. Obesity is a multifactorial health condition, and the target behaviors of the Switch program are only three out of many risk factors [39,42]. If even small changes can be enacted and maintained, the changes may have large effects in the long term [43-45]. It is also encouraging that parents and children perceived that substantial changes had been made during the intervention, and that several positive changes appear to have been maintained 6 months after the program was ended.

There are several strengths of the Switch intervention, including targeting multiple ecological levels and multiple behaviors. A number of school-based intervention studies have been conducted to promote healthy eating and PA [40,41,46,47], but few have also targeted reductions in ST [48,49]. Although often thought of as synonymous constructs, it has been demonstrated that PA and ST have independent effects on risk for overweight [50,51], so both should be included in interventions. Most school-based studies have focused only on curricular or policy changes in physical education and school food service [52-54]. Comprehensive obesity prevention necessitates cooperation from families but this has often been neglected. In their review, Flynn *et al*. [40] highlighted the need for studies that include home and community settings, and the present study found significant changes using measures that focused on behavior in both home and school settings. Another strength of this study was the participatory approach, allowing teachers and parents to adapt the program to their needs. Most previous school-based interventions have utilized tight controls to ensure uniform implementation, but these require frequent staff training and ongoing support [52,55]. That approach is costly and limits sustainability. Our approach was to standardize recommendations to communities, teachers and parents but to allow flexibility in how the materials were used. This approach provides sufficient standardization for statistical analyses while maintaining the external validity of the study. Other studies have demonstrated that this participatory approach has advantages for long-term sustainability [56,57]. A final strength was the employment of multiple informants. Although the effects on FV consumption and ST were in the same direction for both child and parent respondents, the effects of the intervention appeared stronger based on parental report. It is unclear why this was found. It is possible that parents are more accurate reporters of children's habits. Another possibility is that parents are more susceptible to demand characteristics. This does not seem particularly likely, since we would expect this bias in both the experimental and control groups. Furthermore, parents were not uniformly more positive than children: parents were more positive about ST, similar about FV consumption, and less positive about PA (Table 4). Although we cannot determine why the effects appear somewhat different based on the informant, the fact that the effects were in the same direction demonstrates a certain degree of robustness. Furthermore, by obtaining data from multiple informants we were able to minimize the impact of potential measurement problems.

A key challenge in the Switch project was to document and track the extent to which children, parents, and teachers utilized the specific strategies that were provided. Other studies have also reported challenges in getting families to maintain participation in school and/or community projects. Most large-scale, prevention-based interventions targeting families have generally been ineffective because of this limitation [13,14]. In contrast, obesity treatment programs involving families have been shown to be highly effective, if not essential [58]. In fact, an evidence analysis supported by the ADA on youth obesity interventions [26], rated the strength of evidence supporting family involvement with the strongest possible grade (Grade I). It is not clear why family involvement matters but it is likely that parents assume greater responsibility and are more willing to be involved in a clinically based weight loss program than a project aimed at prevention. A major challenge for prevention intervention programs is to determine how to increase the proportion of participants who fully engage in the intervention activities [59,60]. Our results demonstrate that participants who were more involved in the program saw larger improvements in ST and FV than participants that were not as involved. This suggests that the effectiveness of programming would be greatly enhanced if it was possible to get more families to engage fully in the intervention activities.

Community messages were distributed by newspapers, radio advertisements, and billboards, but it is difficult to document the impact that these social-change messages independently had on the outcomes as experimental families were receiving parallel messages from several ecological sources (that is, direct, school, and community). Furthermore, these community-based messages were available to both experimental and control families and, therefore, may have minimized differences between the groups.

Although over 1,300 families participated in the study, a major limitation of this study was the sample size. Statistical analysis of treatment effects was at the school level due to families being nested within schools and randomization at the school level, resulting in low power to detect differences. Future studies should randomize at the classroom or family level to have greater degrees of freedom (although this could lead to greater problems with cross-contamination between experimental and control groups). Another limitation is the potential for social desirability to influence the results. It seems unlikely, however, that only the experimental group would be subject to such desirability biases. To reduce the potential effects of bias and method variance, we used multiple informants. An additional limitation was reliance on pedometers to measure PA. Although it seems preferable to have an objective measure, compliance with the requirements was lower than anticipated. Furthermore, the pedometer captures the total amount of PA and does not provide information on the intensity of PA. It is unknown if the amount of time spent in moderate-to-vigorous PA was altered by the intervention. IHere, it is possible that the total number of steps/day was unchanged yet the time spent in moderate-to-vigorous PA increased. Future studies should use PA monitoring tools that capture both the volume and intensity of PA.

In conclusion, the results indicate that the Switch program produced modest treatment effects for children's FV and ST. It is noteworthy that the effects remained significant in the 6-month follow-up evaluation as this indicates maintenance of these differences over time. Although levels of BMI were not statistically different between groups, the maintenance of behaviors over time may contribute to reduced risks for overweight in the future. The Switch program which targets multiple behaviors through multiple channels offers promise for use in youth obesity prevention.

## Abbreviations

ADA: American Dietetics Association; BMI: body mass index; ES: effect sizes; FV: fruit and vegetable consumption; PA: physical activity; SD: standard deviation; ST: screen time.

## Competing interests

The authors declare that they have no competing interests.

The Switch program is a program of the National Institute on Media and the Family, a non-profit organization. Several of the authors were employed by the Institute to create the program or to conduct the research (DAG, DAW, MW, SS, RC, and KF), or consulted with the Institute on the design (JCE) or analysis (DWR and RAR).

## Authors' contributions

DAG, JCE, DAW, RC, MW, SS, and KF designed the study, established methods and questionnaires, and participated in the coordination of the study. GW provided insight into the conceptual framework of the study. DWR, DAG, and RAR conducted the statistical analyses. All authors read and approved the final manuscript.

## Pre-publication history

The pre-publication history for this paper can be accessed here:



## References

[B1] Wang Y, Beydoun MA (2007). The obesity epidemic in the United States - gender, age, socioeconomic, racial/ethnic, and geographic characteristics: a systematic review and meta-regression analysis. Epidemiol Rev.

[B2] Koplan JP, Liverman CT, Kraak VI (2005). Preventing childhood obesity: health in the balance: executive summary. J Am Diet Assoc.

[B3] Krebs NF, Jacobson MS (2003). Prevention of pediatric overweight and obesity. Pediatrics.

[B4] Baranowski T, Cullen KW, Nicklas T, Thompson D, Baranowski J (2002). School-based obesity prevention: a blueprint for taming the epidemic. Am J Health Behav.

[B5] Prevention CfDCa (1996). Guidelines for school health programs to promote lifelong healthy eating. MMWR Recomm Rep.

[B6] Prevention CfDCa (1997). Guidelines for school and community programs to promote lifelong physical activity among young people. MMWR Recomm Rep.

[B7] Baranowski T, Lin LS, Wetter DW, Resnicow K, Hearn MD (1997). Theory as mediating variables: Why aren't community interventions working as desired?. Ann Epidemiol.

[B8] Stone EJ, McKenzie TL, Welk GJ, Booth ML (1998). Effects of physical activity interventions in youth. Review and synthesis. Am J Prev Med.

[B9] Ritchie LD, Welk G, Styne D, Gerstein DE, Crawford PB (2005). Family environment and pediatric overweight: what is a parent to do?. J Am Diet Assoc.

[B10] Golan M, Crow S (2004). Parents are key players in the prevention and treatment of weight-related problems. Nutr Rev.

[B11] Davison KK, Birch LL (2001). Weight status, parent reaction, and self-concept in five-year-old girls. Pediatrics.

[B12] Davison KK, Birch LL (2002). Obesigenic families: parents' physical activity and dietary intake patterns predict girls' risk of overweight. Int J Obes Relat Metab Disord.

[B13] Nader PR, Baranowski T, Vanderpool NA, Dunn K, Dworkin R, Ray L (1983). The family health project: cardiovascular risk reduction education for children and parents. J Dev Behav Pediatr.

[B14] Nader PR, Sellers DE, Johnson CC, Perry CL, Stone EJ, Cook KC, Bebchuk J, Luepker RV (1996). The effect of adult participation in a school-based family intervention to improve children's diet and physical activity: the Child and Adolescent Trial for Cardiovascular Health. Prev Med.

[B15] Wickrama KA, Lorenz FO, Conger RD (1997). Parental support and adolescent physical health status: a latent growth-curve analysis. J Health Soc Behav.

[B16] Wickrama KAS, Lorenz FO, Conger RD, Elder GH (1998). Parental education and adolescent physical health. J Marriage and Family.

[B17] Katz DL, O'Connell M, Yeh MC, Nawaz H, Njike V, Anderson LM, Cory S, Dietz W (2005). Public health strategies for preventing and controlling overweight and obesity in school and worksite settings: a report on recommendations of the Task Force on Community Preventive Services. MMWR Recomm Rep.

[B18] Booth SL, Sallis JF, Ritenbaugh C, Hill JO, Birch LL, Frank LD, Glanz K, Himmelgreen DA, Mudd M, Popkin BM, Rickard KA, St Jeor S, Hays NP (2001). Environmental and societal factors affect food choice and physical activity: rationale, influences, and leverage points. Nutr Rev.

[B19] Sallis JF, Owen N, Glanz K, Lewis FM, Rimer BK (1997). Ecological models. Health Behavior and Health Education: Theory, Research, and Practice.

[B20] Stokols D, Allen J, Bellingham RL (1996). The social ecology of health promotion: implications for research and practice. Am J Health Promot.

[B21] Welk G (1999). The Youth Physical Activity Promotion Model: A conceptual bridge between theory and practice. Quest.

[B22] Lytle LA, Seifert S, Greenstein J, McGovern P (2000). How do children's eating patterns and food choices change over time? Results from a cohort study. Am J Health Promot.

[B23] Kimm SY, Glynn NW, Kriska AM, Barton BA, Kronsberg SS, Daniels SR, Crawford PB, Sabry ZI, Liu K (2002). Decline in physical activity in black girls and white girls during adolescence. N Engl J Med.

[B24] Huston AC, Donnerstein E, Fairchild H, Feshbach ND, Katz PA, Murray JP, Rubinstein EA, Wilcox BL, Zuckerman DM (1992). Big World, Small Screen: The Role of Television in American Society.

[B25] Gentile DA (2009). Pathological video game use among youth 8 to 18: A national study. Psychol Sci.

[B26] Myers EF (2005). ADA Evidence Analysis Library. J Am Diet Assoc.

[B27] Wetter AC, Goldberg JP, King AC, Sigman-Grant M, Baer R, Crayton E, Devine C, Drewnowski A, Dunn A, Johnson G, Pronk N, Saelens B, Snyder D, Novelli P, Walsh K, Warland R (2001). How and why do individuals make food and physical activity choices?. Nutr Rev.

[B28] Eisenmann JC, Gentile DA, Welk GJ, Callahan R, Strickland S, Walsh M, Walsh DA (2008). SWITCH: rationale, design, and implementation of a community, school, and family-based intervention to modify behaviors related to childhood obesity. BMC Public Health.

[B29] APA (2002). Ethical principles of psychologists and code of conduct. Am Psychol.

[B30] Vincent SD, Pangrazi RP (2002). An examination of the activity patterns of elementary school children. Pediatr Exerc Sci.

[B31] Tudor-Locke C, Pangrazi RP, Corbin CB, Rutherford WJ, Vincent SD, Raustorp A, Tomson LM, Cuddihy TF (2004). BMI-referenced standards for recommended pedometer-determined steps/day in children. Prev Med.

[B32] Malina RM, Maud PJ, Foster C (1995). Anthropometry. Physiological Assessment of Human Fitness.

[B33] Cole TJ, Bellizzi MC, Flegal KM, Dietz WH (2000). Establishing a standard definition for child overweight and obesity worldwide: international survey. BMJ.

[B34] Gentile DA, Walsh DA (2002). A normative study of family media habits. J Appl Dev Psychol.

[B35] Anderson CA, Gentile DA, Buckley K (2007). Violent Video Game Effects on Children and Adolescents: Theory, Research, and Public Policy.

[B36] Gentile DA, Lynch PJ, Linder JR, Walsh DA (2004). The effects of violent video game habits on adolescent aggressive attitudes and behaviors. J Adolesc.

[B37] Department of Health and Human Services and the Centers for Disease Control and Prevention (2005). National YRBS Data Users Manual Atlanta, GA.

[B38] Cohen J (1988). Statistical Power Analysis for the Behavioral Sciences.

[B39] Baranowski T, Mendlein J, Resnicow K, Frank E, Cullen KW, Baranowski J (2000). Physical activity and nutrition in children and youth: An overview of obesity prevention. Prev Med.

[B40] Flynn MA, McNeil DA, Maloff B, Mutasingwa D, Wu M, Ford C, Tough SC (2006). Reducing obesity and related chronic disease risk in children and youth: a synthesis of evidence with 'best practice' recommendations. Obes Rev.

[B41] Story M, Kaphingst KM, French S (2006). The role of schools in obesity prevention. Future Child.

[B42] Eisenmann JC (2006). Insight into the causes of the recent secular trend in pediatric obesity: Common sense does not always prevail for complex, multi-factorial phenotypes. Prev Med.

[B43] Abelson RP (1985). A variance explanation paradox: When a little is a lot. Psychol Bull.

[B44] Rosenthal R (1990). How are we doing in soft psychology?. Am Psychol.

[B45] Glasgow RE, Vogt TM, Boles SM (1999). Evaluating the public health impact of health promotion interventions: the RE-AIM framework. Am J Public Health.

[B46] Brown T, Summerbell C (2009). Systematic review of school-based interventions that focus on changing dietary intake and physical activity levels to prevent childhood obesity: an update to the obesity guidance produced by the National Institute for Health and Clinical Excellence. Obes Rev.

[B47] Summerbell CD, Waters E, Edmunds LD, Kelly S, Brown T, Campbell KJ (2005). Interventions for preventing obesity in children. Cochrane Database Syst Rev.

[B48] Gortmaker SL, Peterson K, Wiecha J, Sobol AM, Dixit S, Fox MK, Laird N (1999). Reducing obesity via a school-based interdisciplinary intervention among youth: Planet Health. Arch Pediatr Adolesc Med.

[B49] Robinson TN (1999). Reducing children's television viewing to prevent obesity. J Am Med Assoc.

[B50] Eisenmann JC, Bartee RT, Wang MQ (2002). Physical activity, TV viewing, and weight in U.S. youth: 1999 Youth Risk Behavior Survey. Obes Res.

[B51] Laurson KR, Eisenmann JC, Welk GJ, Wickel EE, Gentile DA, Walsh DA (2008). Combined influence of physical activity and screen time recommendations on childhood overweight. J Pediatr.

[B52] Hoelscher DM, Feldman HA, Johnson CC, Lytle LA, Osganian SK, Parcel GS, Kelder SH, Stone EJ, Nader PR (2004). School-based health education programs can be maintained over time: results from the CATCH Institutionalization study. Prev Med.

[B53] Sallis JF, McKenzie TL, Conway TL, Elder JP, Prochaska JJ, Brown M, Zive MM, Marshall SJ, Alcaraz JE (2003). Environmental interventions for eating and physical activity: a randomized controlled trial in middle schools. Am J Prev Med.

[B54] Stone EJ, Norman JE, Davis SM, Stewart D, Clay TE, Caballero B, Lohman TG, Murray DM (2003). Design, implementation, and quality control in the Pathways American-Indian multicenter trial. Prev Med.

[B55] Kelder S, Hoelscher DM, Barroso CS, Walker JL, Cribb P, Hu S (2005). The CATCH Kids Club: a pilot after-school study for improving elementary students' nutrition and physical activity. Public Health Nutr.

[B56] Dzewaltowski DA, Estabrooks PA, Johnston JA (2002). Healthy youth places promoting nutrition and physical activity. Health Educ Res.

[B57] Dzewaltowski DA, Estabrooks PA, Welk G, Hill J, Milliken G, Karteroliotis K, Johnston JA (2008). Healthy youth places: a randomized controlled trial to determine the effectiveness of facilitating adult and youth leaders to promote physical activity and fruit and vegetable consumption in middle schools. Health Educ Behav.

[B58] Epstein LH, Myers MD, Raynor HA, Saelens BE (1998). Treatment of pediatric obesity. Pediatrics.

[B59] Dzewaltowski DA, Estabrooks PA, Glasgow RE (2004). The future of physical activity behavior change research: what is needed to improve translation of research into health promotion practice?. Exerc Sport Sci Rev.

[B60] Glasgow RE, Klesges LM, Dzewaltowski DA, Bull SS, Estabrooks P (2004). The future of health behavior change research: what is needed to improve translation of research into health promotion practice?. Ann Behav Med.

